# Comparison of Fe_2_TiO_5_/C photocatalysts synthesized *via* a nonhydrolytic sol–gel method and solid-state reaction method

**DOI:** 10.1039/d0ra07884k

**Published:** 2020-12-09

**Authors:** Qianqian Zhao, Guo Feng, Feng Jiang, Shanfang Lan, Junhua Chen, Mengting Liu, Zuzhi Huang, Jianmin Liu, Qing Hu, Weihui Jiang

**Affiliations:** Department of Material Science and Engineering, Jingdezhen Ceramic Institute Jingdezhen 333000 China jiangfeng@jci.edu.cn jiangweihui@jci.edu.cn +86-798-8499328; National Engineering Research Center for Domestic & Builing Ceramics, Jingdezhen Ceramic Institute Jingdezhen 333000 China fengguo@jci.edu.cn +86-798-8499904; School of Chemical Engineering and Technology, China University of Mining and Technology Xuzhou 221116 China huzf0624@163.com +86-516-83591059

## Abstract

Fe_2_TiO_5_/C photocatalysts were synthesized by a solid-state reaction method (Fe_2_TiO_5_/C_(S)_) and nonhydrolytic sol–gel (NHSG) method (Fe_2_TiO_5_/C_(N)_), where C was introduced by external carbon and *in situ* carbon sources, respectively. The Fe_2_TiO_5_/C_(N)_ photocatalyst with *in situ* carbon has much better photocatalytic degradation efficiency than that of Fe_2_TiO_5_/C_(S)_ synthesized by doping external carbon. The superiorities of *in situ* carbon were demonstrated by SEM, EDS, BET and photoelectrochemical analysis. Compared with Fe_2_TiO_5_/C_(S)_ using external carbon as a carbon source, Fe_2_TiO_5_/C_(N)_ with *in situ* carbon exhibits more uniform elemental distribution, much larger surface area, higher photocurrent density and lower resistivity of interfacial charge transfer. The results show that the introduction of *in situ* carbon *via* the NHSG method more easily promotes the separation of photogenerated electron–hole pairs, owing to the uniformity of the carbon element, thereby improving the photocatalytic activity of the photocatalyst.

## Abstract 2

Two different methods were used to prepare Fe_2_TiO_5_/C photocatalysts, demonstrating the superiorities of *in situ* carbon introduced by a NHSG method.

## Graphical abstract

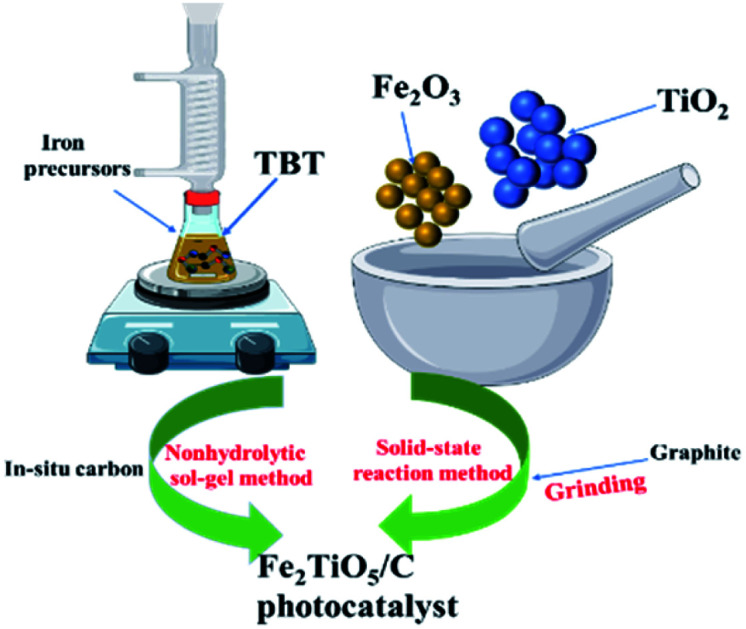

## 1. Introduction

Since the 21^st^ century, environmental pollution, especially water pollution, has gradually become a major problem restricting the survival and development of human society.^1^ Large amounts of dyes are discharged into rivers without any treatment in the textile industry, posing a great threat to water resources.^2^ Much of the recent research has focused on the development of environmental remediation technologies/cleanness of water,^3,4^ which is famous for advanced oxidation processes (AOPs).^5,6^ It is known that inexhaustible solar energy is the most ideal candidate for energy source. Thus, visible-light photocatalysis has received great attention in environmental remediation, which includes organic photocatalysts^7–9^ and inorganic photocatalysts. Xie *et al.* developed visible-light-induced deoxygenative C2-sulfonylation of quinoline N-oxides with sulfinic acids and C(sp^2^)–H/O–H cross-dehydrogenative coupling of quinoxalin-2(1*H*)-ones with alcohols using organic dyes as the catalysts.^7,8^ Though researchers have had some achievements in the field of organic photocatalysis, the inorganic photocatalysts have the merits of high efficiency, simplicity, good repeatability, and easy operation.^10^ Therefore, heterogenous photocatalysis using inorganic materials as photocatalysts is often used to degrade organic pollutants with reactive oxygen species (ROS) and.^11,12^ The increasing public awareness on the advantage of heterogenous photocatalysis has promoted the development of a variety of photocatalytic materials.^13,14^ Fe_2_TiO_5_ is a kind of narrow band gap semiconductor (2.2 eV), which has the strong ability to absorb visible light compared with the traditonal wide band gap semiconductor as diverse as TiO_2_.^15,16^ In addtion, Fe_2_TiO_5_ has the characteristics of low cost, compared with other noble metallic photocatalyst.^17,18^ However, the narrow band gap will lead to the shortcoming of rapid recombination of photoinduce e^−^/h^+^ pairs.^19^ Thus, among the plenty of strategies to solve this problem,^20–22^ carbon doping is one of the most effective routes to modify photocatalyst.^23–26^ Janus *et al.* developed carbon-modified TiO_2_ photocatalyst by exposure of P25 to the ethanol vapor for the carbon deposition.^23^ Zhang *et al.* fabricated a TiO_2_/C photocatalyst using activated carbon as a support by metal organic chemical vapor deposition.^24^ However, the enhancement in the above reports is likely to be the absorption capacity of organic substrates rather than the photocatalytic ability. Besides, the adoption of additional carbon precurors (*e.g.* sucrose, glucose) to prepare carbon-doped metal oxides requires strict preparation conditions and methods.^25,26^ In our recent study, the *in situ* carbon was first introduced by NHSG method into the Fe_2_TiO_5_ photocatalyst.^27^ The Fe_2_TiO_5_/C composite has exhibited superior photocatalytic activity in comparison with Fe_2_TiO_5_. This work aims at exploring the effect of doping methods and uniformity for carbon on the photocatalytic efficiency by the comparison of Fe_2_TiO_5_/C_(N)_ and Fe_2_TiO_5_/C_(S)_. In addition, three synthetic routes are designed by using different iron precursors to investigate the reason why the Fe_2_TiO_5_ can be synthesized at lower temperature by NHSG method.

## 2. Materials and experimental methods

### 2.1 Reagents and materials

Graphite (99.95%) and Fe_2_O_3_ (99.5%) were obtained from Aladdin. TiO_2_ (99%) was purchased from Macklin. Iron(iii) chloride (FeCl_3_, >98%) and basic ferric acetate (FeOH(CH_3_COO)_2_, 99.99%, metals basis) were purchased from Aladdin. Iron(iii) ethoxide (Fe(OC_2_H_5_)_3_, >99%, metals basis) was obtained from Shanghai Myrell Chemical Technology Co., Ltd. Tetra-*n*-butyl titanite (Ti(OC_4_H_9_)_4_, chemical pure) and ethanol (C_2_H_5_OH, >99.7%) were supplied by Sinopharma Chemical Reagent Co.,Ltd. Starting materials are reagent special grade.

### 2.2 Preparation of Fe_2_TiO_5_/C

#### 2.2.1 Solid-state reaction method

**Fig. 1:**
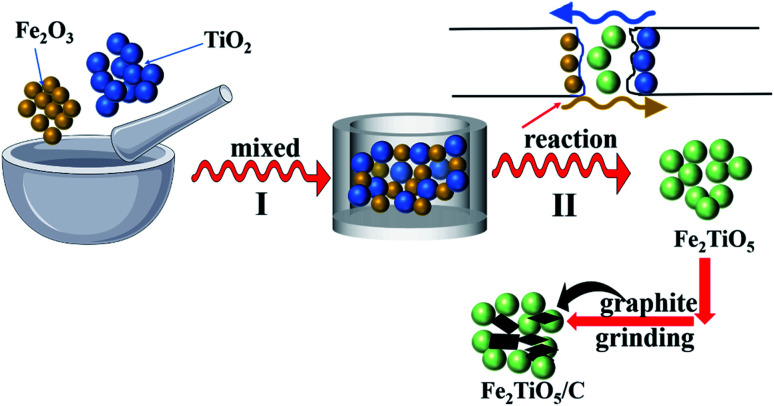
The preparation process of Fe_2_TiO_5_/C_(S)_*via* solid-state reaction method.

First, 0.04 mol Fe_2_O_3_ was mixed with 0.08 mol TiO_2_ using agate mortar. The mixtures reacted with each other to generate Fe_2_TiO_5_ at high temperature. A certain amount of graphite was mixed with as-prepared Fe_2_TiO_5_ powder through grinding with different grinding time (0.5 h, 1 h). The final mixtures were denoted as Fe_2_TiO_5_/C_(S)_ (0.5 h) and Fe_2_TiO_5_/C_(S)_ (1 h) (Fig. 1).

#### 2.2.2 NHSG method

**Fig. 2:**
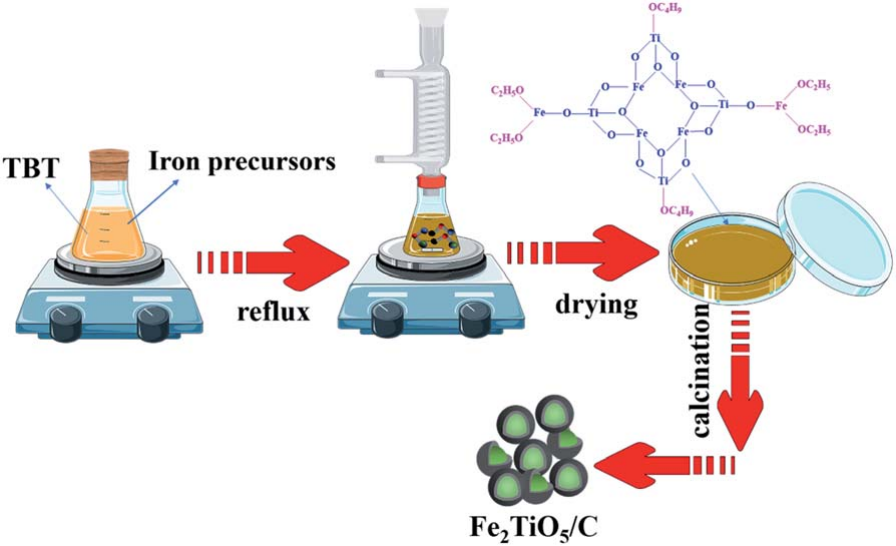
The preparation process of Fe_2_TiO_5_/C_(N)_*via* NHSG method.

The Fe_2_TiO_5_/C_(N)_ was prepared by a NHSG method. Firstly, iron precursors was dissolved in anhydrous ethanol. Iron precursors inculde Iron(iii) ethoxide (Fe(OEt)_3_), basic ferric acetate (FeOH(CH_3_COO)_2_) and ferric chloride (FeCl_3_). The preparation process was shown in Fig. 2. Tetra-*n*-butyl titanite was added to above solution in the molar ratio of Fe : Ti = 1 : 1 with vigorous stirring at 40 °C for 1 h to generate the precursor sol. Afterwards, the sol was refluxed with magnetic stirring in oil bath at 80 °C for 24 hours with the formation of wet gel. Next, the xerogel was acquired after dring wet gel was dried at 110 °C for 6 hours. The xerogel was then calcined to obtain Fe_2_TiO_5_/C_(N)_, where C was provided by the carbon-containing precursors.

### 2.3 Photoelectrochemical measurement

The photoelectrochemical properties of the prepared samples were measured on a CHI760E electrochemical station using a standard three-electrode cell. A Pt sheet was employed as the counter electrode. A saturated calomel electrode and the as-prepared photocatalyst acted as the reference electrode and working electrode, respectively. The working electrode is prepared by the following process: first, 10 mg as-prepared powder were evenly dispersed in 2 mL ethanol solution by ultrasound for 30 minutes, then 30 μL suspension sampled was dropped onto the ITO-coated glass and dried at room temperature for photoelectrochemical measurement. Thereinto, the transient photocurrent response measurement was carried out in 0.5 M Na_2_SO_4_ aqueous solution under a 300 W xenon light irradiation/using a 300 W Xe lamp as illumination source. Electrochemical Impedance Spectroscopy (EIS) was conducted in 0.5 M K_3_[Fe(CN)_6_] solution chosen as electrolyte solution and was carried out under static condition with scanning frequencies from 100 kHz to 0.01 Hz and amplitude of 5 mV.

### 2.4 Evaluation of visible light photocatalytic activity

The visible-light catalytic activities of the obtained Fe_2_TiO_5_/C prepared by two methods were investigated using MB as the degradation object at ambient temperature under visible light irradiation. 0.25 g of as-prepared sample was dispersed in 100 mL (50 mg L^−1^) dye solution. Before the photoreaction started, the suspension kept stirring in dark for 30 min to reach the adsorption–desorption equilibrium between the photocatalyst and the MB aqueous solution. The suspension under magnetic stirring was illuminated during exposure to visible light (>400 nm) using a 300 W Xe lamp as light source. 5 mL aliquots were withdrawn at certain time intervals for analysis. The photocatalytic degradation performance was evaluated by measuring the residual concentration of the simulating pollutant as a function of light time with an ultraviolet–visible Lambda850 spectrophotometer (PerkinElmer Instrument Company, America).

## 3. Characterizations

DSC-TG/DTA-TG analysis was conducted on a simultaneous thermal analyzer (NETZSCH STA449C). The crystal phases of the as-prepared products were characterized by a D8 Advance X-ray diffractometer at 40 kV and 30 mA using CuKα radiation source. The morphological observations were carried out by a field-emission scanning electron microscope (JEOL JSM-6700E) equipped with an energy-dispersive X-ray spectroscopy (EDS). For identifying the chemical bonds of xerogel, Fourier transform infrared spectroscopy (FTIR) were recorded between 400 and 4000 cm^−1^ using a Nicolet 5700 spectrometer. Raman spectra was recorded from 100 cm^−1^ to 2000 cm^−1^ on a Renishaw Laser Confocal Raman Spectrometer(in *Via*, England). Brunauer–Emmett–Teller (BET) specific surface area of as-synthesized samples were evaluated by isothermal N_2_ adsorption with an ASAP 2020 surface area analyzer of Micromeritics.

## 4. Results and discussion

### 4.1 Fe_2_TiO_5_/C synthesized by solid-state reaction method

**Fig. 3:**
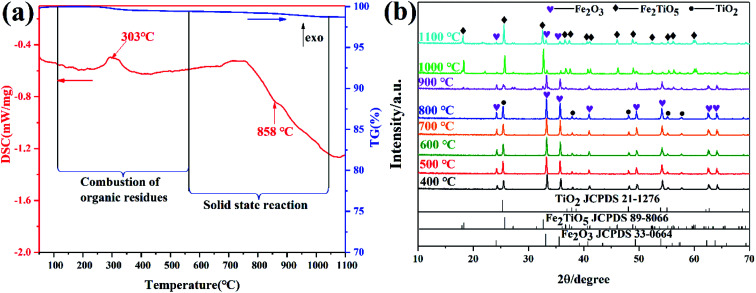
DSC-TG curves of the mixture of Fe_2_O_3_ and TiO_2_ (a); XRD patterns of samples calcined at varying temperatures by solid-state reaction method (b).

**Fig. 4:**
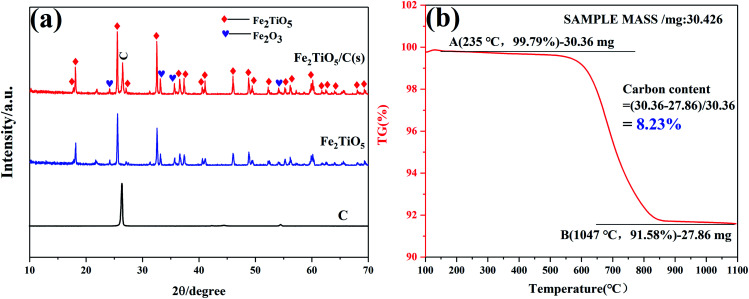
XRD patterns of graphite, Fe_2_TiO_5_ and Fe_2_TiO_5_/C_(S)_ (a), TG of Fe_2_TiO_5_/C(s) at O_2_ atomosphere (b).

The DSC and TG curves of the Fe_2_O_3_ and TiO_2_ mixture, shown in Fig. 3a, exhibit two major peaks and one mass regions from 50 °C to 1100 °C, respectively. Fig. 3b displays the XRD patterns of samples calcined at varying temperatures by solid-state reaction method. The exothermic peak centered at 303 °C can be ascribed to the combustion of residual organics in the mixtures, corresponding to 0.586% mass loss in Fig. 3a. The endothermic peak located at 879 °C in the DSC curve is related with the phase transition,^28^ owing to the first occurrence of Fe_2_TiO_5_ phase from 800 °C to 900 °C in the XRD patterns. The solid-state reaction is shown in formula (1). Moreover, it should be pointed out that some diffraction peaks indexed to hematite Fe_2_O_3_ (JCPDS 33-0664) still exist at 1100 °C as shown in Fig. 3b, which indicates the difficulty in obtaining pure phase Fe_2_TiO_5_ for solid-state reaction method. In addtion, it undoubtedly increases industrial preparation costs traced to the higher energy by solid-state reaction method. Ultimately, Fe_2_TiO_5_/C_(S)_ is obtained by adding graphite into Fe_2_TiO_5_ prepared by solid-state reaction method, as shown in the XRD pattens (Fig. 4a). In order to test the carbonaceous content in sample, thermogravimetry (TG) cuve was obtained at O_2_ atmosphere, as shown in Fig. 4d. According to the mass of the sample tested and the final weight loss in the test process, the carbonaceous content of Fe_2_TiO_5_/C_(S)_ is calculated to be 8.23 wt%.1
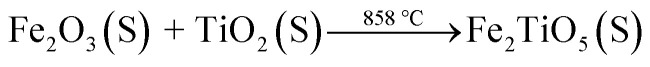


### 4.2 The effect of iron precursors on the synthesis of Fe_2_TiO_5_

NHSG method is a liquid-phase synthesis method in organic solvents which overcomes the disparity in the reaction rates of the different sol–gel precursors in the traditional hydrolytic sol–gel method (HSG). In addtion, NHSG route is easier to achieve uniform mixing at the atomic level, promoting the preparation of homogeneous mixed metal oxide system.^29^ Especially, metal organic compounds and organic solvents including a plenty of carbon-containing groups are usually used as raw materials, providing the necessary carbon source for the synthesis of *in situ* carbon doped Fe_2_TiO_5_. There are usually three routes for the synthesis of heterometallic compounds by NHSG method.^30^

(1) Alkyl chloride elimination route:M′(OR)_*n*′_ + MCl_*n*_ → (OR)_*n*′−1_M′–O–M(Cl)_*n*−1_ + RCl,where R represents C_2_H_2*n*+1_ (alkyl) group.

(2) Ether elimination route:M′(OR′)_*n*′_ + M(OR)_*n*_ → (OR′)_*n*′−1_M′–O–M(OR)_*n*−1_ + R′OR,where R′ and R stand for C_2_H_2*n*+1_ (alkyl) group.

(3) Ester elimination route:M′(OR′)_*n*′_ + M(OZ)_*n*_ → (OR′)_*n*′−1_M′–O–M(OZ)_*n*−1_ + R′OZ,where Z refers to an CH_3_CO (acetyl) group.

**Fig. 5:**
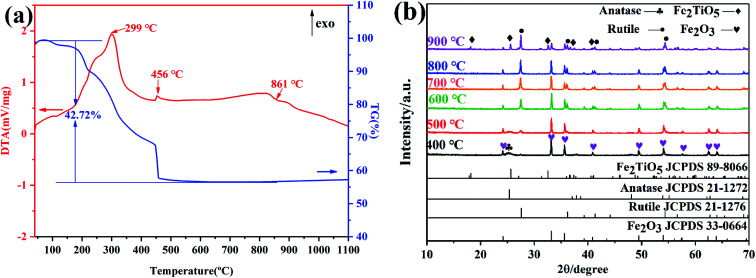
DTA-TG curves of xerogel using FeCl_3_ as iron precursor (a); XRD patterns of samples calcined at different temperatures (b) by NHSG method.

Here, in order to verify the feasibility of the above three routes in the synthesis of Fe_2_TiO_5_, FeCl_3_, FeOH(CH_3_COO)_2_ and Fe(OEt)_3_ were used as iron precursors, respectively. Fig. 5a presents the DTA-TG curves of xerogel using FeCl_3_ as iron precursor. The first two exothermic peaks at 299 °C and 456 °C are ascribed to the combustion of residual carbon and the transition of anatase to rutile, corresponding to a weight loss of 42.72%. The last exothermic peak centered at 889 °C shows the reaction of Fe_2_O_3_ and TiO_2_ to form Fe_2_TiO_5_ accompanied almost no mass loss, which is also cofirmed by the later XRD results. Fig. 5b shows the XRD patterns of samples calcined at different temperatures using FeCl_3_ as iron precursor by NHSG method. It can be seen that the weak Fe_2_TiO_5_ diffraction peak appeared until 900 °C. The phase transition process during calcination is shown below.2
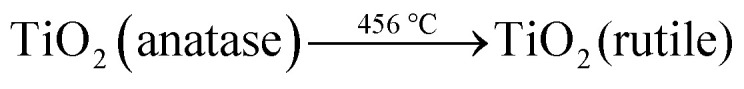
3
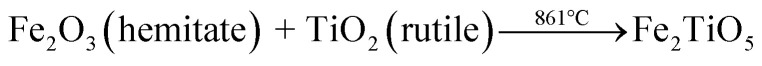


**Fig. 6:**
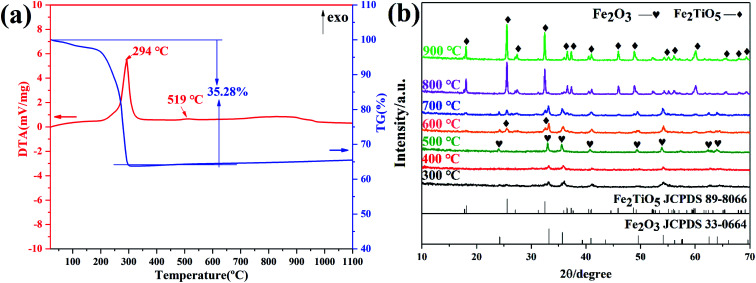
DTA-TG curves of xerogel using FeOH(CH_3_COO)_2_ as iron precursor (a); XRD patterns of samples calcined at varying temperatures (b) by NHSG method.

Fig. 6a presents the DTA-TG curves of xerogel using FeOH(CH_3_COO)_2_ as iron precursor. A sharp exothermic peak in the DTA curve at 294 °C is related with the carbonization of organic residues and the volatilization with burning of part residual organic group,^27^ corresponding to a mass loss of 35.28% in the TG curve. In addtion, the phase transition from amorphous Fe_2_TiO_5_ to crystallized Fe_2_TiO_5_ contributes to the small exotherm at 519 °C (shown in formula (4)), which is supported by XRD results in Fig. 6b.4
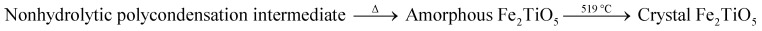


**Fig. 7:**
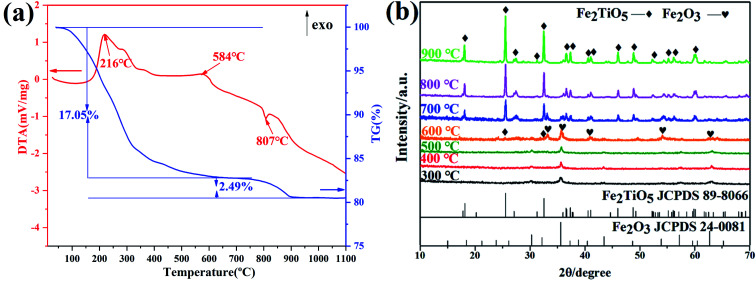
DTA-TG curves of xerogel using Fe(OEt)_3_ as iron precursor (a); XRD patterns of samples calcined at varying temperatures (b) by NHSG method.

Fig. 7 shows DTA-TG curves of xerogel and XRD patterns of samples calcined at varying temperatures *via* NHSG method using Fe(OEt)_3_ as iron precursor. In the first stage, a 17.05% weight decrease due to with the carbonization of organic residues and the volatilization with burning of part residual organic group, corresponding to an exothermic reaction at 216 °C. In the second stage between 500 °C and 600 °C, an exothermic peak at 584 °C originates from the formation of crystalline Fe_2_TiO_5_ modification from the amorphous form. XRD analysis showed that Fe_2_TiO_5_ crystallization started at lower temperatures. The whole reaction process during heatment can be summarized in formula (5).5
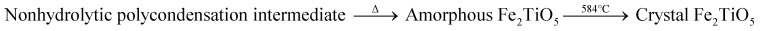


**Fig. 8:**
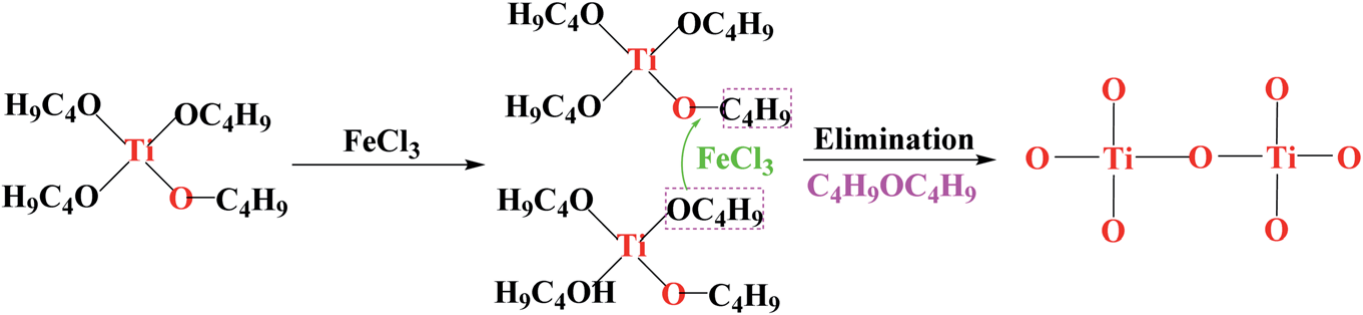
Schematic diagram of the proposed mechanism for the formation of Ti–O–Ti linkages.

**Fig. 9:**
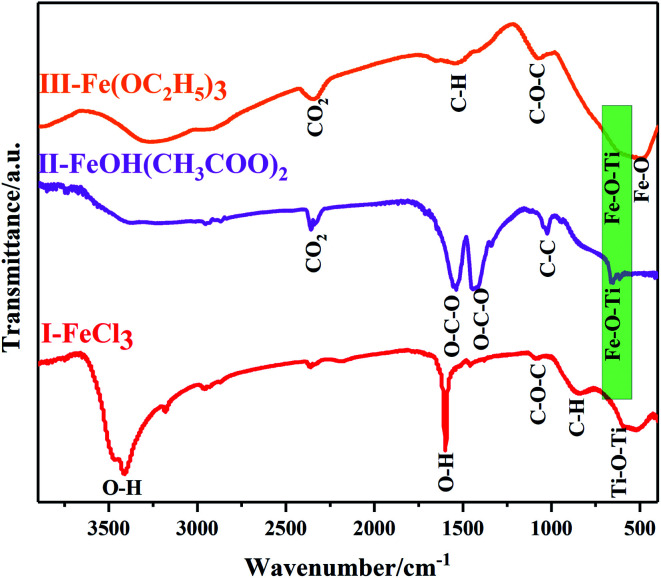
FT-IR spectra of xerogels prepared with different iron precursors *via* NHSG method.

Furthermore, Fourier transform infrared spectrometer (FTIR spectrometer) was used to identify the chemical bonds and functional groups of xerogels with different iron precursors. From the FTIR spectrum of I–FeCl_3_, the absorption peak appear at the range of 500–600 cm^−1^, belonging to Ti–O–Ti stretching vibrations.^31,32^ At the same time, the absence of Fe–O–Ti bond (629 cm^−1^) suggests the failure of reaction route (1), which is because that FeCl_3_, as Lewis acid, can promote homogeneous polycondensation to form Ti–O–Ti linkages along with the elimination of C_4_H_9_OC_4_H_9_, as shown in Fig. 8.^33^ At the same time, the characteristic peak of ether appears at 1100 cm^−1^ verify the existence of C_4_H_9_OC_4_H_9_.^34^ Moreover, the FTIR spectrum of xerogel using FeOH(CH_3_COO)_2_ as iron precursor is shown in Fig. 8-II. The FTIR spectrum of II-FeOH(CH_3_COO)_2_ showed two obvious absorption peaks at 1428 cm^−1^ and 1546 cm^−1^, which is attributed to the symmetric stretching vibration of and the anti-symmetric stretching vibration of the O–C–O bond, respectively.^35^ In addition, it is worth noting that Fe–O–Ti bond appears in the III-FeOH(CH_3_COO)_2_,^36^ which demonstrates the occurrence of heterogeneous polycondensation in gel using FeOH(CH_3_COO)_2_ as iron precursor, as shown in reaction eqn (7). Meanwhile, in the FTIR spectrum of II-Fe(OEt)_3_, a weak absorption peak located at 629 cm^−1^ is ascribed to the stretching vibration of Fe–O–Ti bond, indicating the occurrence of reaction eqn (8). The peak at 1100 cm^−1^ shows stretching vibrations of C–O–C bond in C_4_H_9_OC_2_H_5_.^37^ The 1525 cm^−1^ band is attributed to C–H vibrational modes.^38^ The existence of C–H bonds indicates that the xerogel contains some residual organic functional groups, on account of the incomplete polycondensation substitution reaction.^39^ It is helpful for the formation of *in situ* doped C during post-heating treatment (Fig. 9).

6 Ti(OC_4_H_9_)_4_ + FeOH(CH_3_COO)_2_ → (H_9_C_4_O)_4−*x*_Ti–O–Fe(OH)(CH_3_COO)_2−*x*_ + *x*C_4_H_9_OOCCH_3_, (0 < *x* < 2)

7 Ti(OC_4_H_9_)_4_ + Fe(OC_2_H_5_)_3_ → (H_9_C_4_O)_4−*y*_Ti–O–Fe(OC_2_H_5_)_3−*y*_ + *y*C_4_H_9_OC_2_H_5_, (0 < *y* < 3)

In conclusion, Fe_2_TiO_5_ can be synthesized at the lower temperature (800 °C) using FeOH(CH_3_COO)_2_ and Fe(OEt)_3_ as iron precursors on the account of uniform mixing at the atomic level and the formation of Fe–O–Ti linkages. However, the C element mass fractions of FeOH(CH_3_COO)_2_ and Fe(OEt)_3_ were calculated to be 25.14% and 37.70% accroding to the formula (8), thus, Fe(OEt)_3_ containing more carbon-based groups can provide more *in situ* carbon for the synthesis of Fe_2_TiO_5_/C. In the following, Fe_2_TiO_5_/C prepared with Fe(OEt)_3_ as the iron precursor *via* NHSG method was compared with the Fe_2_TiO_5_/C prepared by the solid-state reaction method.8
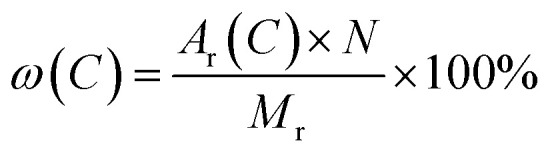
where *ω* refers to element mass fraction, *A*_r_, *M*_r_ and *N* stand for the relative atomic mass, relative molecular mass and atomic number, respectively.

### Microstructure analysis

**Fig. 10:**
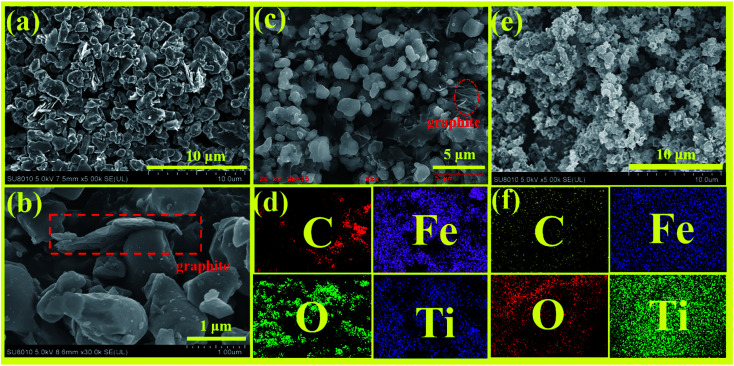
FE-SEM images with different magnification (a–c); EDS mapping (d) of Fe_2_TiO_5_/C_(S)_; FE-SEM images (e); EDS mapping (f) of Fe_2_TiO_5_/C _(N)_.

Fig. 10a–c show the SEM images of Fe_2_TiO_5_/C prepared by solid-phase method with different magnifications. It can be seen that the sample has irregular bulk distribution morphology and the two-dimensional graphite sheets as carbon source only were only stacked in local area in SEM image. In order to evaluate the uniformity of elemental distribution, EDS elemental mapping analysis of Fe_2_TiO_5_/C prepared by solid-state reaction method is presented in Fig. 10d. The mapping result shows that the C element is not evenly distributed in sample, which matches well with the corresponding SEM image. Fig. 10e and f display the SEM image and elemental mapping of Fe_2_TiO_5_/C_(N)_, respectively. Fe_2_TiO_5_/C_(N)_ composite is composed of a large number of hollow spherical particles. C, Fe, O, Ti elements disperse evenly in the sample, indicating the successful incorporation of C element into Fe_2_TiO_5_ hollow spherical structure. In addition, the hollow spherical structure permits light to be reflected multiple times inside the hollow spheres, thereby promoting the utilization of the incident light.^40^

### Form and content of *in situ* carbon introduced by NHSG

**Fig. 11:**
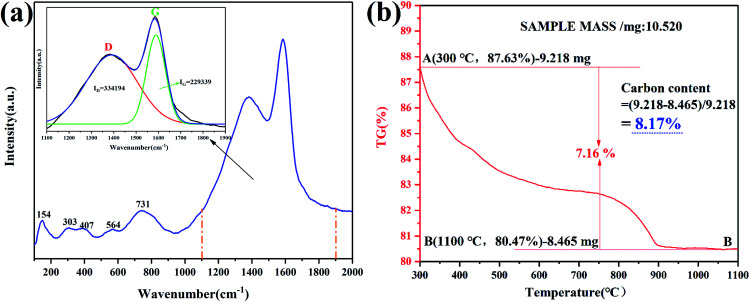
Raman spectra and main peak fitting (inset) (a), TG of Fe_2_TiO_5_/C_(N)_ (b).

Fig. 11a shows the Raman spectroscopy of Fe_2_TiO_5_/C_(N)_. From 100–1000 cm^−1^, several broad bands could be attributed to Fe_2_TiO_5_ (154, 303, 407, 564, 731 cm^−1^). The band at 1384 cm^−1^ is associated with the vibrations of carbon atoms with dangling bonds for the in-plane terminations of disordered graphite and is labeled as the D-band, and the band at 1589 cm-1 (G-band) (corresponding to the E_2g_ mode) was closely related to the vibration in all sp^2^ bonded carbon atoms in a 2-dimensional hexagonal lattice. The intensity ratio of the D to G band (*I*_D_/*I*_G_) is calculated to be 1.457 according to the integral area values of D and G peaks obtained by peak fitting, indicating the *in situ* carbons contain a lot of defects. Defects are easier to capture photogenerated electrons, thereby inhibiting the recombination of photogenerated electrons/holes.^41^ According to TG curve in Fig. 11b, the carbonaceous content was calculated into 8.17 wt%, which is very close to carbonaceous content of Fe_2_TiO_5_/C_(s)_ (8.23 wt%).

### Specific surface area analysis

It is known to all that the smaller specific surface area (SSA_BET_) is regarded as the other limitations in semiconductor photocatalysis because the photocatalysis occurs at the surface of photocatalyst. Sample with larger specific surface area can offer more active sites, thereby promoting the photocatalytic reaction. The SSA_BET_ of Fe_2_TiO_5_/C_(S)_ and Fe_2_TiO_5_/C_(N)_ is calculated to be 3.5100 m^2^ g^−1^ and 195.5153 m^2^ g^−1^, respectively. The SSA_BET_ value of the latter is almost 56 times that of the former, indicating Fe_2_TiO_5_/C_(N)_ is more conducive to facilitating the mass-transport and charge-transfer.^42^

### Photocatalytic activity analysis

**Fig. 12:**
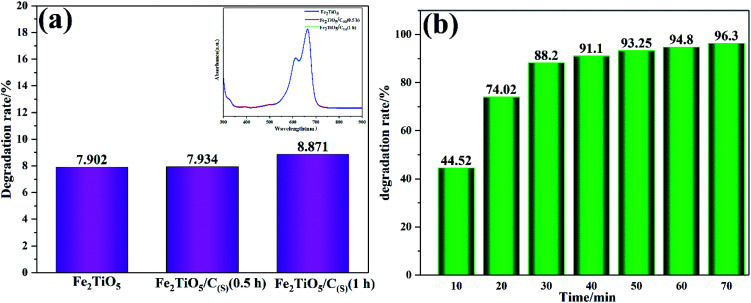
The photocatalytic degradation efficiency and UV-vis absorption spectra (inset) of MB in the presence of Fe_2_TiO_5_; Fe_2_TiO_5_/C_(S)_ (0.5 h); and Fe_2_TiO_5_/C_(S)_ (1 h) prepared by solid-state reaction method (a); irradiation time dependence of the photodegradation rate of MB by Fe_2_TiO_5_/C_(N)_ (b).

Fig. 12 shows the photodegradation efficiency of Fe_2_TiO_5_/C composites towards MB dye prepared by two different methods. Fig. 12a shows the photodegradation rate of Fe_2_TiO_5_ and Fe_2_TiO_5_/C prepared by solid-state reaction method with visible-light irradiation. Thereinto, Fe_2_TiO_5_/C_(S)_ (0.5 h) and Fe_2_TiO_5_/C_(S)_ (1 h) refer to the mixture of Fe_2_TiO_5_ and graphite obtained with different grinding time. MB was photodegraded by 7.902%, 7.934%, and 8.871% in the presence of Fe_2_TiO_5_, Fe_2_TiO_5_/C_(S)_ (0.5 h) and Fe_2_TiO_5_/C_(S)_ (1 h), respectively. The results of photodegradation experiments show that the increasing grinding time is conducive to the improvement of photocatalytic performance, indicating the uniformity of carbon is an important factor for enhancing photocatalytic performance of sample. However, a little change in the UV absorption spectra in the upper right corner in Fig. 12 and the inconspicuous enhancement from 7.902% to 8.871%, reveals that it is unsatisfactory to promote the catalytic performance of samples by adding additonal carbon. Consequently, the uniform *in situ* carbon is introduced into Fe_2_TiO_5_ by containg-carbon precursors *via* NHSG method for the purpose of improving photocatalytic activity. The Fe_2_TiO_5_/C_(N)_ photocatalyst has the higher photodegradation rate (88.2%) in comparison with the Fe_2_TiO_5_/C_(S)_ photocatalyst (8.871%) exposed to visible light for 30 min in Fig. 12b. And the dye can be almost completely decomposed by the Fe_2_TiO_5_/C_(N)_ under the visible light irradiation within 70 min, indicating that the photocatalyst has excellent visible light photocatalytic performance.

### Separation of photoexcited electrons and holes

**Fig. 13:**
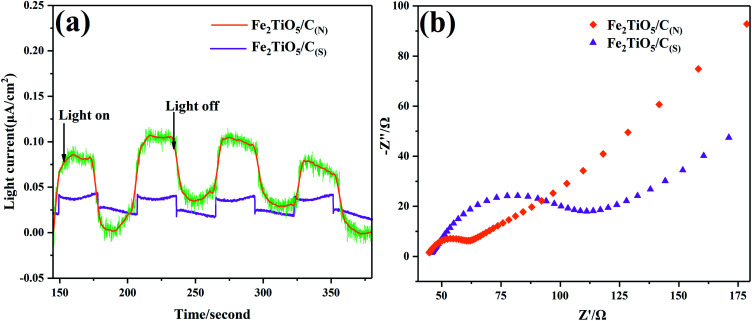
Transient photocurrent responses under visible-light irradiation (a), EIS spectra of Fe_2_TiO_5_/C_(N)_ and Fe_2_TiO_5_/C_(S)_ (b).

Photocurrent response analysis has been widely recognized as an effective means to evaluate the separation ability of photogenerated carriers. When light energy is used to excite the material, the valence band electrons are excited and transition to the conduction band. Under the strong electric field, the conduction band electrons will move directionally to form a current, that is, a photo-generated current. As shown in Fig. 13a, the photocurrent–time curves (*I*–*T* curves) were performed for several on–off cycles of visible-light irradiation (*λ* > 420 nm). The photocurrent of Fe_2_TiO_5_/C_(N)_ electrode is significantly higher than that of Fe_2_TiO_5_/C_(S)_. In general, when the optical radiation energy is absorbed by the semiconductor material to generate a photocurrent, a higher photocurrent response indicates better charge separation performance.^43^ Therefore, the NHSG method is more conducive to promoting the separation of photo-generated carriers, owing to the excellent electron transportation ability of the uniform *in situ* carbon introduced by NHSG, which is in good agreement with the photocatalytic activity test results shown in Fig. 12.

Electrochemical Impedance Spectroscopy (EIS) offers another strong evidence for exploration of interfacial charge transfer behaviors. During the electrochemical impedance test of photocatalytic materials, the electrochemical impedance spectroscopy (EIS) obtained generally consists of an “arc” and a “tail”, as shown Fig. 13b. The “arc” in the high-frequency and low-resistance region is mainly dominated by the charge transfer. The “tail” in the low-frequency high-resistance region is mainly controlled by the mass transfer. Therefore, *R*_ct_ (charge transfer resistance) is generally determined by the arc radius. It is clearly shown that the arc radius on EIS Nyquist plot of Fe_2_TiO_5_/C _(N)_ with *in situ* carbon is much smaller than that of Fe_2_TiO_5_/C _(S)_ prepared by adding extra carbon, which indicates the Fe_2_TiO_5_/C_(N)_ presents higher charge separation rate. The smaller arc radius corresponds to the lower *R*_ct_ value and weaker impedance facilitating the separation of photoinduced electron–hole pairs, which is consistence with the results of photocurrent response analysis.^44^

## 5. Conclusion

This work makes a systematical comparison of Fe_2_TiO_5_/C photocatalysts synthesized by NHSG method and solid-state reaction method. In conclusion, the Fe_2_TiO_5_/C_(N)_ with *in situ* carbon as carbon source has higher catalytic efficiency under visible-light irradiation than that of Fe_2_TiO_5_/C_(S)_ with the extra carbon. The *in situ* carbon plays the important role in enhancing the visible-light catalytic ability. Most *in situ* carbon evenly distributed in the sample which greatly facilitates electron transport and promotes the separation of photogenerated electrons and holes. Photoelectrochemical measurements have been utilized to evaluate the separation ability of photogenerated carriers. Compared with Fe_2_TiO_5_/C_(s)_ with addition carbon, Fe_2_TiO_5_/C_(N)_ with *in situ* carbon has more photocurrent and weaker resistance, indicating the effective separation of photogenerated electrons and holes in the photocatalytic system of Fe_2_TiO_5_/C_(N)_.

## Conflicts of interest

There are no conflicts to declare.

## Supplementary Material
